# Hierarchical Ag nanostructures on Sn-doped indium oxide nano-branches: super-hydrophobic surface for surface-enhanced Raman scattering

**DOI:** 10.1039/c8ra01510d

**Published:** 2018-04-06

**Authors:** Kyungchan Min, Kyoung Soon Choi, Wook Jin Jeon, Dong Kyu Lee, Sein Oh, Jouhahn Lee, Jae-Young Choi, Hak Ki Yu

**Affiliations:** Dept. of Materials Science and Engineering & Dept. of Energy Systems Research, Ajou University Suwon 16499 Korea hakkiyu@ajou.ac.kr +82(0)31-219-1613 +82(0)31-219-1680; The Advanced Nano Surface Research Group, Korea Basic Science Institute Daejeon 34144 Korea; School of Advanced Materials Science & Engineering, School of Advanced Institute of Nanotechnology (SAINT), Sungkyunkwan University Suwon 16419 Korea jy.choi@skku.edu

## Abstract

Herein, we fabricated a super-hydrophobic SERS substrate using Sn-doped indium oxide (Indium-tin-oxide: ITO) nano-branches as a template. ITO nano-branches with tens of nanometer diameter are first fabricated through the vapor–liquid–solid (VLS) growth to provide roughness of the substrate. 10 nm thickness of Ag thin film was deposited and then treated with the post-annealing process to create numerous air-pockets in the Ag film, forming a hierarchical Ag nanostructures. The resulting substrate obtained Cassie's wetting property with a water contact angle of 151°. Compared to the normal hydrophobic Ag nanoparticle substrate, increase of about 4.25-fold higher SERS signal was obtained for 7 μL of rhodamine 6G aqueous solutions.

## Abstract 2

Herein, we fabricated a super-hydrophobic SERS substrate using Sn-doped indium oxide (Indium-tin-oxide: ITO) nano-branches as a template.

## Graphical abstract

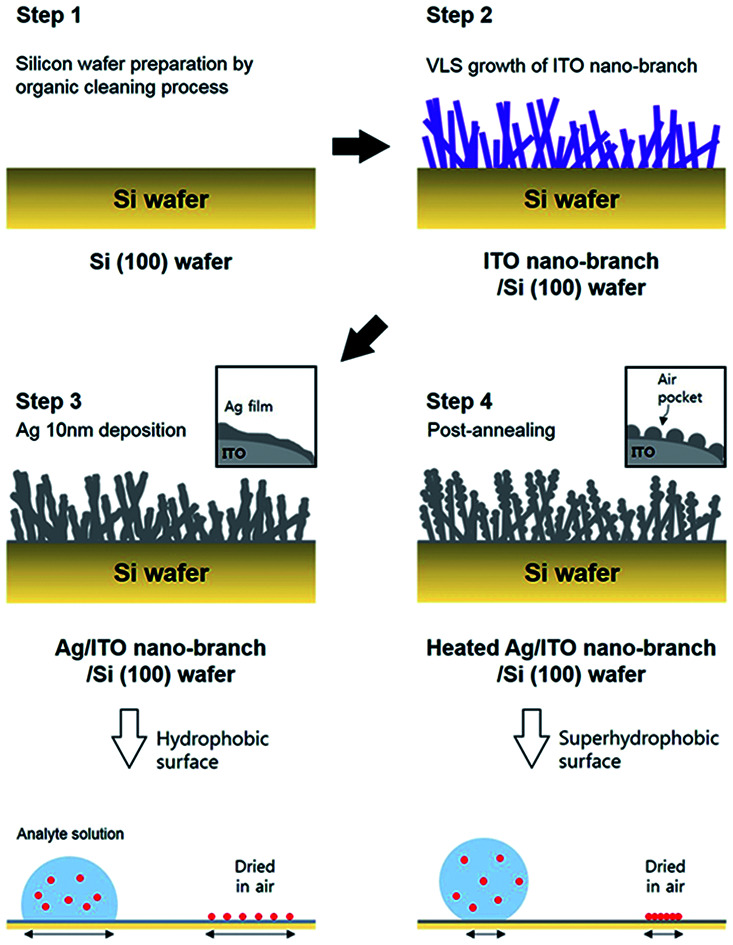

## Introduction

Surface-enhanced Raman scattering (SERS) is one of the most powerful techniques for probing ultra-trace molecules in solutions. Due to its high sensitivity towards SERS, it is widely used in various fields, such as biology, chemistry, materials science, and medicine. Since the discovery of surface enhanced Raman spectroscopy in 1977, various fabrication methods for SERS substrate have been studied for higher sensitivity.^1,2^ It is notable that there are more possibilities for development depending on various fabrication and measurement methods. The most common material for SERS substrate is metal nanoparticles such as silver and gold because these kind of metals causes the enhancement of Raman signal by means of localized surface plasmon resonance (LSPR).^2–6^ When the incident monochromatic light, may in the visible, near infrared, or near ultraviolet, illuminates the metal substrate, LSPR occurred in narrow gap, also called “hot spots”, between nanoparticles such as silver or gold.^7–14^ The electromagnetic field coupled with the analyte molecules absorbed on this gap is dramatically amplified at a certain excitation wavelength resulting in an increase of the Raman signal as high as (*E*^4^/*E*^4^_0_). In this regard, the efficient of SERS is affected by a shape and size of metal nanoparticles.

Many different kinds of metal nanostructures, such as nanorods, nanospheres, nanocubics, or nanoparticles were designed to create hot spots. The usual strategy for fabrication of substrate has just been focusing on creating hotspots uniformly in the metal film *via* the complicated instruments, like electron beam lithography. However, the usual SERS substrate fabricated through these kind of methods has a predominantly hydrophilic surface making the analyte solutions spread over the whole surface, and therefore the SERS effect is limited despite high enough density of hotspots. If it is possible to make analytes be condensed on the specific area of the surface, much higher enhancement will be obtained with the same concentration of analytes. One of the best breakthrough to overcome this limitation is the super-hydrophobic surfaced SERS substrate.^15–20^ Super-hydrophobic surface can reduce the contact area between the surface and the analyte solutions by about 4 to 9 times comparing to the usual hydrophilic substrate.

The usual method to improve hydrophobicity for SERS substrate is to deposit metal thin film and reduce the surface energy with self-assembled monolayer (SAM) of the hydrophobic materials. However, these kind of surface treatment require a few hours of processing time and even take place overnight, and especially, it is possible that the Raman scattering of the hydrophobic materials which consists of C–H chain structures on the metal surface interferes with the Raman scattering of the target molecules. For an accurate analytic result, it is necessary to create super-hydrophobic SERS substrate without any other surface treatment including SAM coating.

Herein, we demonstrate the facile fabrication method of ITO/Ag super-hydrophobic SERS substrate with a hierarchical structure obtained through the post annealing process. This SERS substrate can be inducing the condensation effect of highly diluted analyte molecules. The ITO branches which acted as a template for improved roughness of substrate was grown *via* VLS method by the electron beam evaporation and followed by a deposition of Ag thin film for reducing surface energy. Because these ITO nano-branches can be grown at large area through the simple vapor deposition method, our fabrication method does not need the complicated techniques, such as lithography,^21^ nano-imprinting,^22^ laser ablation,^23^*etc.* Ag (due to broad range of LSPR wavelength between 300–1200 nm compare to Au, Ag was used in our study) thin films on the ITO nano-branches tend to form nano-particles due to screening effect by ITO nano-branches, resulting in hierarchical structure which is optimized for generating strong Raman scattering effect. Finally, the thermal annealing process of the substrate was performed to achieve super-hydrophobicity (using air-pocket between Ag nano-particles) and the effect of this annealing process on SERS performance is demonstrated experimentally.

## Experimental

### Synthesis of super-hydrophobic ITO/Ag SERS substrate

**Fig. 1:**
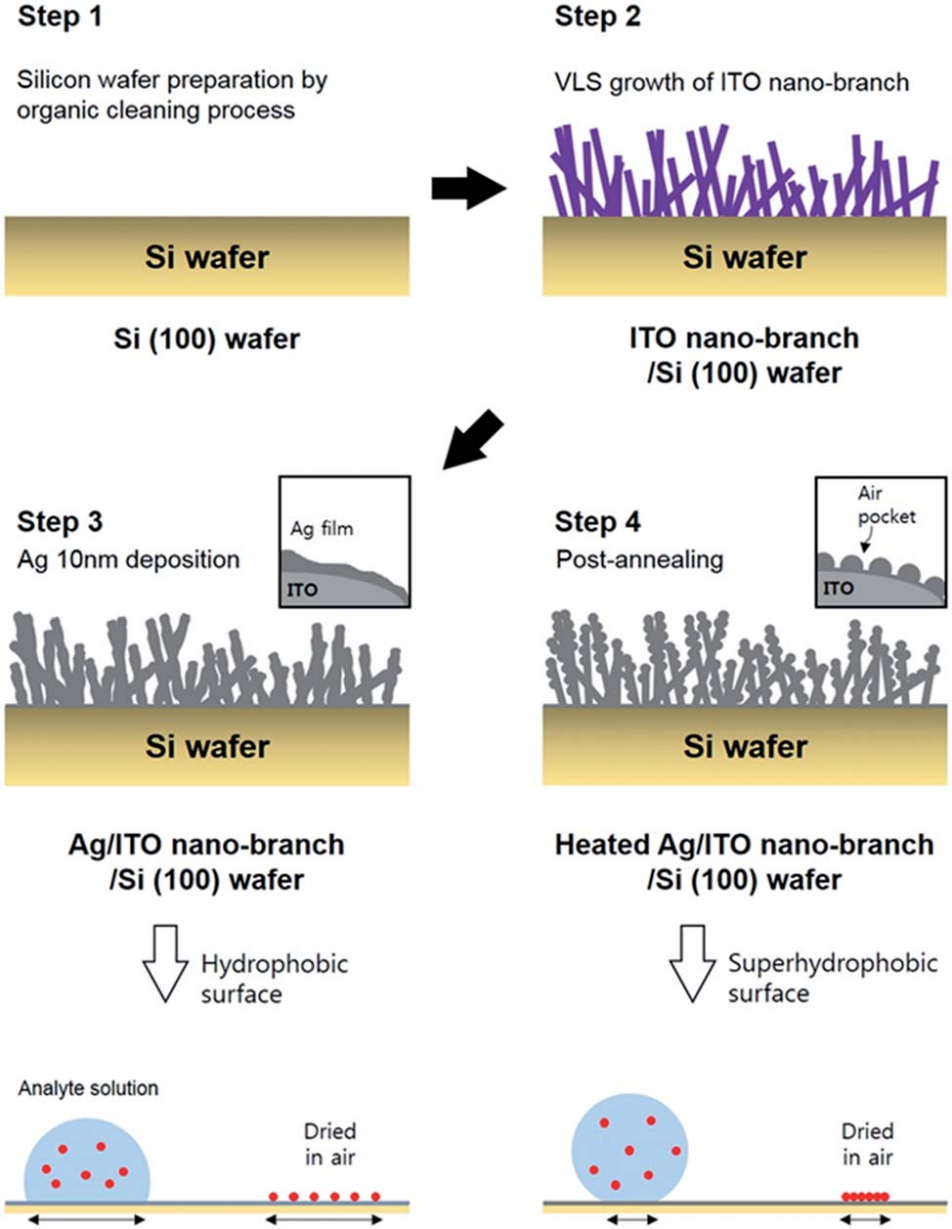
Schematic illustration for fabrication of ITO/Ag superhydrophobic SERS substrate. (Step 1) Silicon (100) wafer preparation by organic cleaning process; (Step 2) VLS growth of ITO nano-branches by electron beam evaporation; (Step 3) the deposition of Ag thin film on the ITO template; (Step 4) the annealing process for a super-hydrophobic surface, resulting in a condensation effect of analyte molecules.

First, the p-type silicon (100) wafer was ultrasonically bathed with acetone, isopropyl alcohol, and deionized water for 10 minutes respectively and then flowed by nitrogen gas. Each layer (ITO nano-branches and Ag film) was prepared by e-beam evaporation method. ITO nano-branches were grown at 350 °C and below 1.0 × 10^−5^ torr of base pressure. Total thickness of ITO nano-branches layer was about 800 nm. Subsequently, thin Ag film was deposited using the same equipment at the room temperature in a high vacuum of 2.0 × 10^−6^ torr. The deposition rate of the Ag was sustained near 0.01 nm s^−1^ until the thickness reaches 10 nm. Finally, the substrate was annealed in a low vacuum condition at 300 °C for 10 minute resulting in a super-hydrophobic surface of Ag. The annealing process was conducted in a low vacuum of 5.0 × 10^−3^ torr. The whole experimental process is represented schematically in Fig. 1.

### Measurement

The morphology of the sample was characterized by field emission scanning electron microscopy (FE-SEM, JEOL JSM-6700F). 1 μL of water droplet was dropped carefully on the substrate and observed by optical contact angle meter at ambient temperature. X-ray photoemission spectroscopy (XPS) was carried out in an AXIS Ultra DLD model (KRATOS, U.K.) at the Korea Basic Science Institute. XPS spectra were obtained at a base pressure of 2.0 × 10^−10^ torr at 300 K with a monochromatic Al Kα line at 1486.69 eV. At last, Raman spectra was obtained by high resolution Raman spectrometer (HORIBA, Jobin Yvon, LabRam HR Evolution). Rhodamine 6G (R6G) dye was chosen as the analyte molecules for the SERS measurement. The concentration of the R6G solutions is varied from 100 ppm to 10 ppb corresponding to 2.1 × 10^−4^ M and 2.1 × 10^−8^ M, respectively. All of the Raman measurement was carried out using 7 μL of R6G solutions after the droplet of solutions is evaporated in air, room temperature. The Raman scattering was acquired with a 633 nm He–Ne laser as excitation source, 17 μW of laser power, and the illuminating spot size was about 0.89 μm in a diameter through a 100× objective. The acquisition time is 5 s with 2 accumulation times.

## Results and discussion

**Fig. 2:**
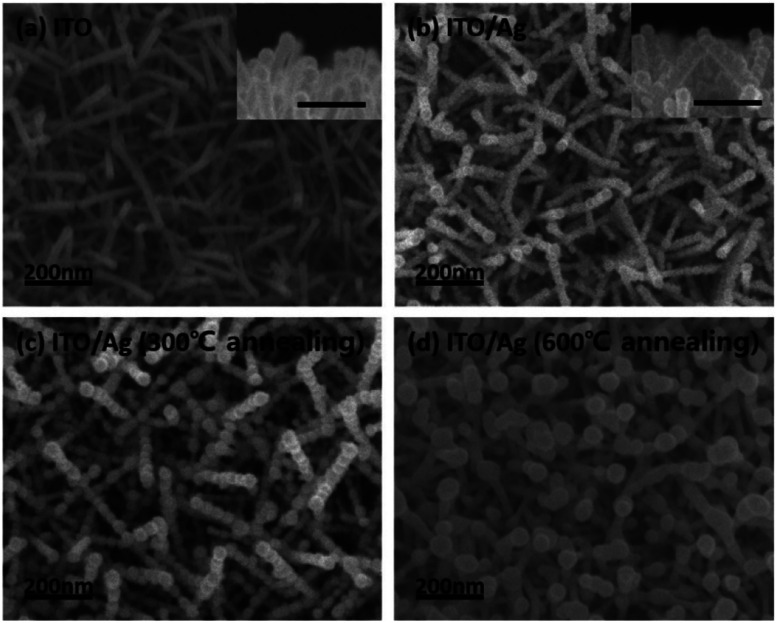
The SEM images of the (a) ITO nano-branches and Ag thin film deposited on the ITO template at different annealing temperature of (b) as-dep, (c) 300 °C, and (d) 600 °C.

ITO nano-branches were used for our super-hydrophobic SERS substrate template. ITO nano-branches were densely grown *via* VLS method by e-beam evaporation with ITO source onto the Si (100) wafer. It has been well investigated that ITO can be clearly grown in rod form at the temperature above the melting point of indium metal, which acts as a self-catalyst in the VLS method.^24–26^Fig. 2a shows the SEM images of the successfully grown ITO nano-branches which are top-view image and cross section view image, respectively. The diameter of the ITO nano-branches are uniformly distributed to about 30 nm. It is quite notable result for the super-hydrophobic SERS substrate because thickness uniformity not only affects the reproducibility of the hydrophobicity but also has an important role in the amplification of the electromagnetic field occurring in the hot spots. Successfully grown ITO nano-branches can act as a template, serving nanoscale roughness which is necessary for super-hydrophobic surface. And then, for a strong enhancement on the substrate surface, Ag is deposited after ITO nanobranches because of their resonance wavelength. That is, Ag is more suitable material than ITO in terms of the LSPR wavelength. The spectrum of the Ag LSPR ranges from the 300 nm to 1200 nm, while that of ITO does at about 3000 nm.^27^ As deposited Ag over-layer would be forming hierarchical structure and acting as an antenna amplifying the Raman laser. A very thin 10 nm of Ag was deposited onto the substrate for the lower surface energy.^28–30^ Ag thin film should be thinly deposited for the following two reasons. Firstly, it can retain nanoscale roughness of substrate. As mentioned above, ITO template has a nanoscale roughness, so the morphology of the substrate will be changed significantly if lots of Ag are coated onto the ITO surface. Secondly, thin Ag film is easy to aggregate during the deposition resulting a hierarchical structure. As shown in the Fig. 2b, thin Ag film clearly covered both tops and sides of the ITO surface forming a hierarchical structure, which can contain numerous plasmonic hotspots due to screening effect. Most ITO surfaces are covered by the silver, which means, the analyte molecules are getting little contacted with the ITO due to the Ag over-layer. A lots of Ag nanoparticles was created on the surface with very narrow gap acting as an antenna for the electromagnetic field enhancement. Ag film coated on the ITO surface was then annealed in a low vacuum conditions to protect against the oxidation on the Ag surface. As the annealing progressed, Ag film was mildly agglomerated improving the roughness of the surface. This hierarchical Ag structure is maintained when it is heated to 300 °C, however, when it is heated above 600 °C, the structure is deformed by silver agglomeration as shown in Fig. 2c and d.^31,32^

**Fig. 3:**
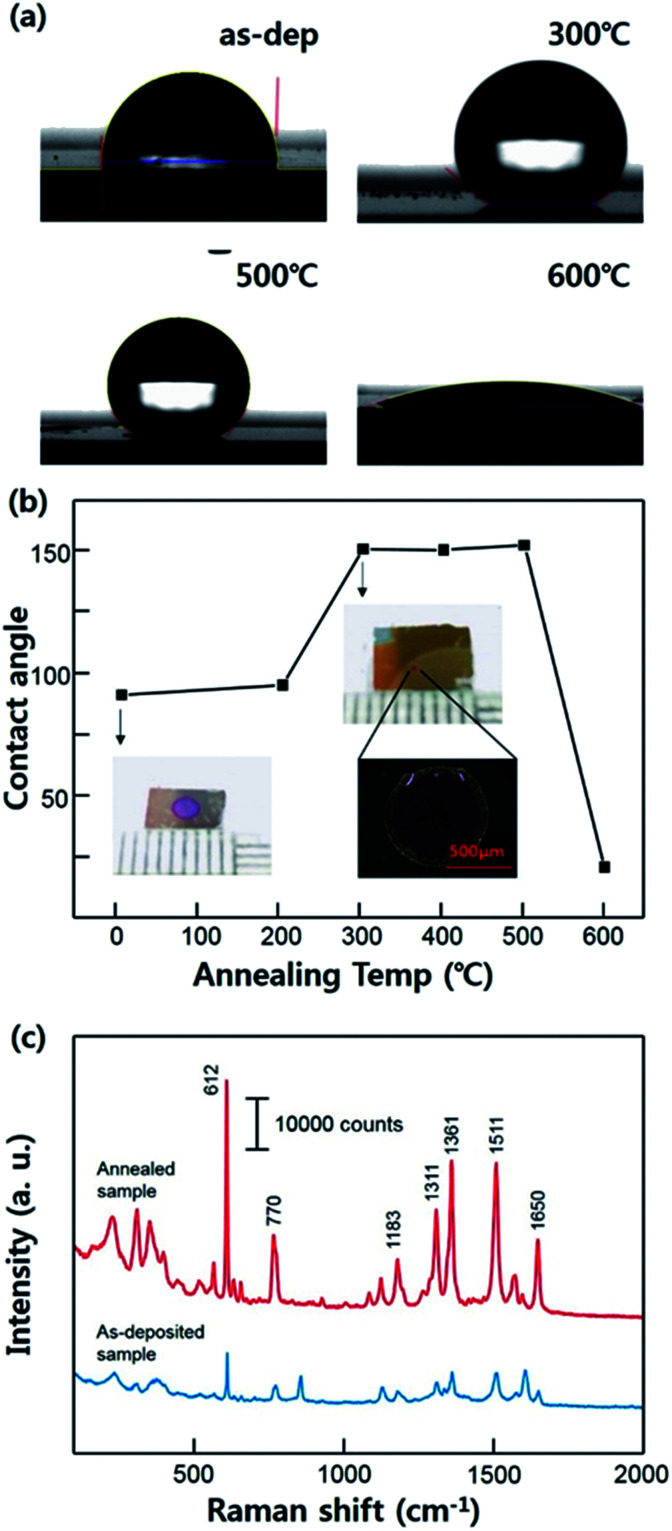
(a) The lateral images of the water droplet on the substrates with different annealing temperatures. As-deposited ITO/Ag sample showing normal hydrophobic contact angle of 92°. The sample annealed at 300 °C and 600 °C showing a contact angle of 151° and 20°, respectively. (b) Dependence of the contact angle with the annealing temperature. (c) The Raman spectra of the R6G using an as-deposited sample and an annealed sample.

We measured the contact angle with the different annealing conditions of the substrates as shown in Fig. 3a. These series of the images clarify the effect of the annealing condition on Ag surface showing an alteration of the contact angle. As-deposited Ag film exhibits normal hydrophobic contact angle about 92°. The hydrophobicity of the substrates was similar until the annealing temperature was 200 °C. However, the contact angle increased after the substrate was annealed at 300 °C, reaching about 151°, and this improved super-hydrophobicity was maintained stably until 500 °C (SERS measurements were carried out using the sample which was annealed at 300 °C). This result demonstrates that the super-hydrophobic Ag surface can be acquired without any other hydrophobic material interfering with the Raman signal of the analyte molecules. However, the contact angle was decrease to about 20° when the temperature rises to 600 °C. This result seems to be that dramatically change of the surface property is caused by the excessive agglomeration of the Ag films. In this case, Ag films lost its hierarchical nanostructure, becoming more flat surface relatively. The dependence of the contact angle on the annealing temperature is shown in Fig. 3b. The images of R6G mark after the evaporation are also shown demonstrating a condensation effect. The Fig. 3c clarified the SERS enhancement depending on the hydrophobicity on the surface. On the super-hydrophobic surface, the analyte molecules was concentrated in the specific area smaller than that on the as-deposited surface due to its condensation effect. This Raman spectra indicate that the super-hydrophobic surface facilitates the higher signal amplification even with a small volume of droplet.

**Fig. 4:**
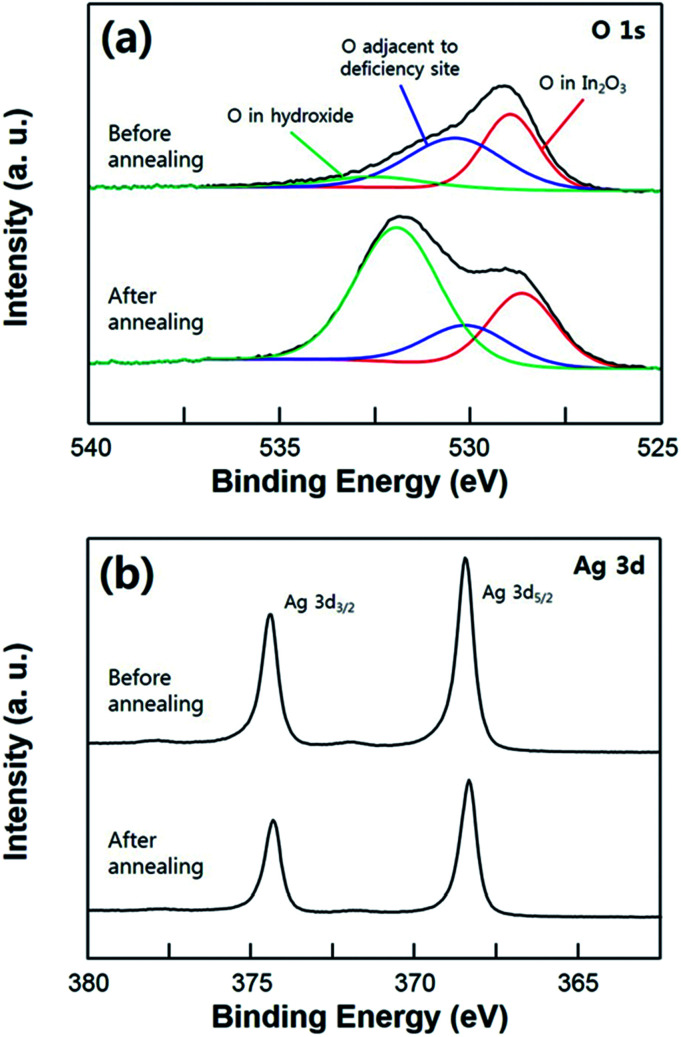
XPS spectra of the O 1s (a) and Ag 3d (b) core level comparing the as-deposited (hydrophobic) sample and the annealed sample (super-hydrophobic).

In Fig. 4, the super-hydrophobicity of the substrate achieved by the annealing process is demonstrated through the XPS analysis. According to these XPS results, the Ag film was undergoing a mild agglomeration with the post annealing process resulting in a hierarchical structural surface. As shown in Fig. 4a, the O 1s core level spectra on the ITO surface was deconvoluted into three peak with binding energy at 533, 531.8, and 530.7 eV, corresponding to the oxygen in the In(OH)_*x*_, In_2_O_3_ (adjacent to the oxygen deficiency site), and In_2_O_3_ (without deficiency), respectively.^33–35^ The O 1s peak with the low binding energy at 530.7 eV, which is assigned to the lattice oxygen in the In_2_O_3_, did not change with the annealing process. On the other hand, the significant increase of the O 1s peak at 533 eV is observed due to the composition with the hydroxyl group originated from the moisture in the air. As the sample annealed, the oxygen atoms in the In_2_O_3_ (adjacent to the oxygen deficiency) are slightly reduced. Reduced oxygen atoms can form dangling bonds on top of the surface, which can increase the oxygen peak of OH group.^34^ This result indicates that the ITO surface was covered by Ag film in the as-deposited sample, and then after the post annealing process, exposed to the ambient air due to the agglomeration of the Ag film. Although the usual hydroxide species have a hydrophilic property, the annealed ITO/Ag substrate exhibits the super-hydrophobic surface. This result can be explained through the heterogeneous wetting property on the surface, also known as the Cassie–Baxter state. That is, liquid droplet placed onto the substrate was not in direct contact with ITO surface due to the presence of the air-pocket on the rough surface. The reduced intensity of Ag 3d peak is also caused by the morphological change of the Ag film which has undergone an agglomeration. And the XPS shift of the Ag 3d can be explained by some of the surface contamination, not by the surface oxidation of the silver. Change of the chemical state causes the increase of the oxidation number, therefore, broadening or shoulder peak of the XPS peak necessarily have to be observed. Especially, the broadening of an Ag 3d peak was not observed after the annealing process, which means that there is no transition of chemical state of Ag atoms at the surface.^36,37^ These XPS analysis results demonstrate that the post annealing process causes an agglomeration of the Ag film forming a hierarchical structure with nano-scaled roughness on the surface, which is resulting in an increase of the hydrophobicity on the surface.

**Fig. 5:**
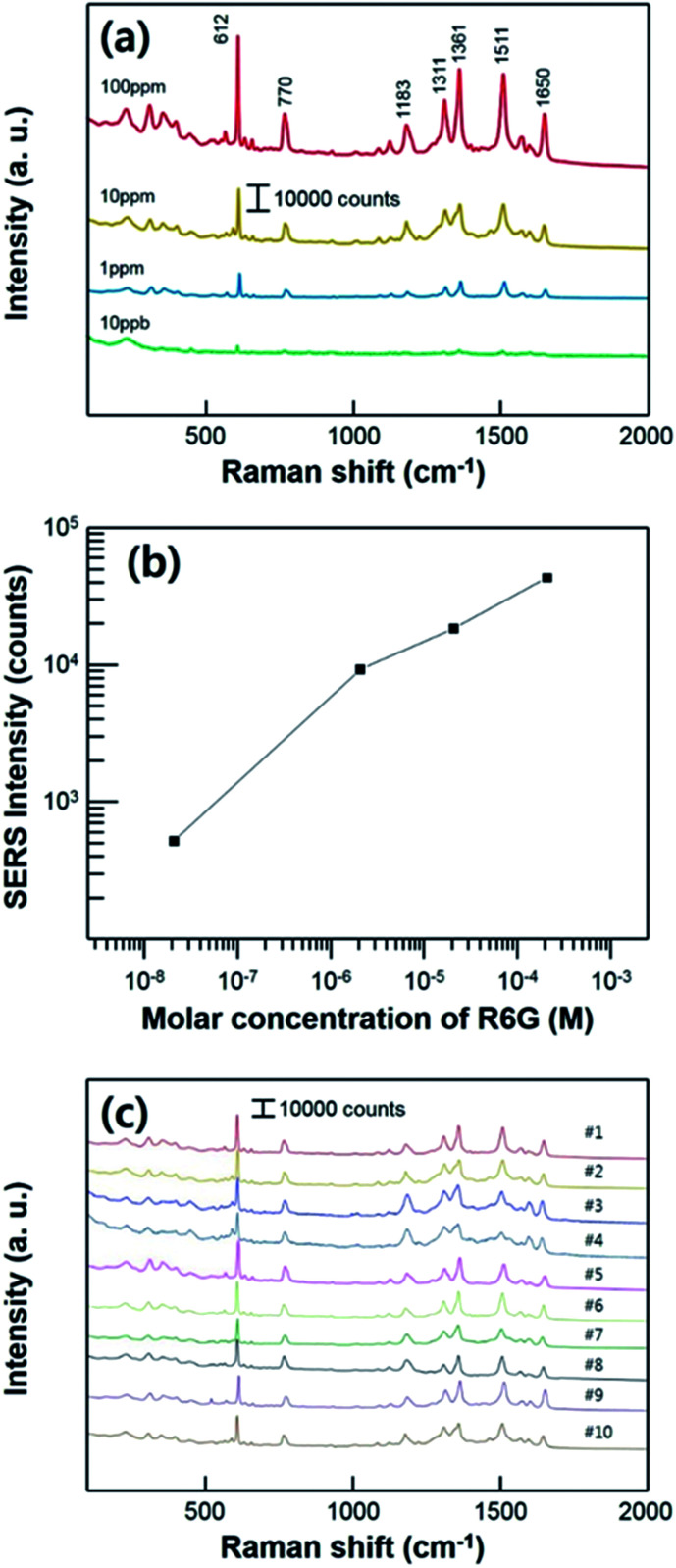
(a) The Raman spectra of the R6G with different concentrations of solution ranging from the 100 ppm (2.1 × 10^−4^ M) to 10 ppb (2.1 × 10^−8^ M) using the super-hydrophobic SERS substrate. (b) Raman intensities at 610 cm^−1^ for the R6G. (c) Raman spectra of R6G solution using the super-hydrophobic SERS substrate during ten cycles of Raman measurement.

To measure the SERS performance of the substrate, we prepared the different concentrations of Rhodamine 6G (R6G) solution which is widely used as a target molecules for SERS measurement. All of the Raman measurements were conducted after the evaporation of R6G solutions, using the super-hydrophobic SERS substrate. We used a low power of laser with short acquisition time (as mentioned in experimental section) to avoid a Raman peak saturation caused by a too high intensity. Fig. 5a shows the Raman spectra of the substrate varying the R6G concentration ranging from 100 ppm to 10 ppb which corresponding to 2.1 × 10^−4^ M and 2.1 × 10^−8^ M, respectively. The characteristic bands of R6G are clearly shown from all of the different concentration of solutions at 612 cm^−1^ (C–C–C in-plane bending), 770 cm^−1^ (C–H out-of-plane bending), 1183 cm^−1^ (C–H in-plane bending), 1311 cm^−1^ (aromatic C–C stretching), 1361 cm^−1^ (aromatic C–C stretching), 1511 cm^−1^ (aromatic C–C stretching), and 1650 cm^−1^ (aromatic C–C stretching) in the measurement range.^38^ The intensities of characteristic Raman peak decreased as the proved R6G concentration decreased. In this report, we performed Raman measurement by lowering the concentration of the solution to 10 ppb (2.1 × 10^−8^ M). Fig. 5b shows quantitative comparison of the SERS intensities at 610 cm^−1^ with different concentrations ranging and a linear correlation was acquired. Raman peaks of the R6G were shown until the concentration of 10 ppb (2.1 × 10^−8^ M), including the characteristic Raman peak at 610 cm^−1^ which intensity is estimated to about 500 counts. Fig. 5b shows the concentration dependence of Raman signal at 610 cm^−1^. This linear proportional relation implies that the SERS substrate has a potential about the SERS sensitivity for ultra-low concentration and ability for the quantitative analysis. And also, this result confirms the stability of the signal fluctuation on our substrate. The fabricated substrate has high reusability and stability for the super-hydrophobic SERS effect with repeated SERS tests as shown in Fig. 5c. In the middle of every measurement procedure, evaporated R6G analyte was washed and sonicated in isopropyl-alcohol bath for 10 min. After the substrate completely dried, 7 μL of R6G solutions droplet with 10 ppm (2.1 × 10^−5^ M) of concentration was used for SERS measurement. The super-hydrophobicity of the substrate remained unchanged during ten cycles of repeated Raman measurement. The entire Raman spectrum indicates that our substrate can be used repeatedly.

## Conclusions

In conclusion, this study suggested a novel fabrication method for super-hydrophobic SERS substrate. The Ag thin film was deposited onto the ITO nano-branch, followed by the annealing process for rendering a super-hydrophobicity. It is notable that the super-hydrophobicity of our ITO/Ag SERS substrate was acquired by post annealing process. After the annealing process at the 300 °C, the Ag film is mildly agglomerated forming a hierarchical structure. Due to this morphological change of Ag surface, our substrate does not need a surface treatment with hydrophobic materials. As-prepared ITO/Ag SERS substrate has a super-hydrophobic wetting surface with 151° of contact angle. As a result, a droplet of the Rhodamine 6G solution is successfully condensed on a specific area of the surface, and enhanced SERS signal can be obtained. Especially, our super-hydrophobic SERS substrate can be reused after a washing of substrate. This paper suggests a facile method for efficient Raman sensor, which overcome the limitation of the conventional devices.

## Conflicts of interest

There are no conflicts to declare.

## Supplementary Material
